# In Vitro Effects of Mentha suaveolens, Rosmarinus officinalis, and Lippia citriodora Essential Oils on Sperm Motility, Vitality, and DNA Fragmentation

**DOI:** 10.7759/cureus.105627

**Published:** 2026-03-21

**Authors:** Chaimaa Hilali, Moncef Benkhalifa, Chafika Nadifi, Mohamed Kettani Halabi, Nouama Bouanani, Noureddine Louanjli, Fatima Azzahra Lahlou

**Affiliations:** 1 Oncopathology, Center for Doctoral Studies (CEDoc) Cancer Biology and Environment Laboratory, Mohammed VI University of Health Sciences (UM6SS), Casablanca, MAR; 2 Reproductive Medicine and Biology, Périnatalité et risques toxiques (PériTOX) Laboratory (Unité Mixte de Recherche - I01), Centre Universitaire de Recherche en Santé (CURS), University of Picardy Jules Verne, Department of Reproductive Medicine and Biology, Amiens University Hospital, Amiens, FRA; 3 Medicine, Oncopathology, Cancer Biology and Environment Laboratory, Mohammed VI Faculty of Medicine, Mohammed VI University of Health and Sciences (UM6SS), Casablanca, MAR; 4 Pharmacy, Mohammed VI Faculty of Pharmacy, Mohammed VI University of Health and Sciences (UM6SS), Casablanca, MAR; 5 Hematology, Faculty of Medicine, Mohammed VI University of Health Sciences (UM6SS), Casablanca, MAR; 6 Reproductive Biology, In Vitro Fertilization Center (IRIFIV), Iris Clinic, Casablanca, MAR; 7 Reproductive Biology, Labomac In Vitro Fertilization Center and Clinical Laboratory Medicine, Casablanca, MAR; 8 Biochemistry, Faculty of Medicine, Mohamed VI University of Health Sciences (UM6SS), Casablanca, MAR

**Keywords:** asthenozoospermia, dna fragmentation index, essential oil supplementation, in vitro, male infertility, normozoospermia, phytotherapy, sperm chromatin decondensation index, sperm motility, sperm vitality

## Abstract

Introduction and objective

Phytotherapy offers a promising approach for treating male infertility by harnessing the bioactive properties of various medicinal plants. Studies have shown that plant extracts rich in antioxidants and anti-inflammatory compounds can improve sperm quality, particularly by reducing oxidative stress, a key factor in decreased motility and DNA integrity of spermatozoa. Our study aimed to investigate how *in vitro *supplementation with *Mentha suaveolens *(apple mint), *Rosmarinus officinalis *(rosemary), and *Lippia citriodora *(lemon verbena)affects human sperm motility and vitality. We also evaluated their influence on sperm DNA integrity by measuring the DNA fragmentation index (DFI) and the sperm chromatin decondensation index (SDI). In addition, this study investigated the concentration-dependent effects of these essential oils using an *in vitro *experimental approach and included the separate analysis of normozoospermic and asthenozoospermic samples in order to better assess their potential impact on sperm quality.

Materials and methods

We tested different concentrations of the three essential oils (*Mentha suaveolens, Rosmarinus officinalis, Lippia citriodora) *from 10 µg/µl to 80 µg/µl on normozoospermic (n=120) and asthenozoospermic (n=120) sperm samples *in vitro *to assess their impact on sperm parameters, including motility, vitality, DNA fragmentation index, and sperm chromatin decondensation index.

Results

Our results revealed a strong association between essential oils and sperm parameters. A significant improvement in motility was observed for normozoospermic and asthenozoospermic samples at a concentration of 10 µg/µl in the two essential oils *Mentha suaveolens *and *Rosmarinus officinalis,* and at a concentration of 40 µg/µl for the essential oil *Lippia citriodora*. However, a significant difference was noted in sperm vitality. Furthermore, both the DNA fragmentation index and the sperm chromatin decondensation index showed statistically significant differences in both samples (p < 0.001).

Conclusion

These data suggest that essential oil supplementation could be a promising approach to improving sperm quality and potentially enhancing male fertility.

## Introduction

Infertility is a worldwide public health concern, defined as the failure of a couple to achieve pregnancy after 12 months of regular, unprotected sexual intercourse [1]. It affects an estimated 8-12% of couples of reproductive age, with prevalence influenced by geographic and socio-economic factors [2]. Male infertility accounts for nearly 50% of cases, with multiple etiologies including genetic, hormonal, environmental, and lifestyle factors [3]. It can be caused by several factors, including abnormalities in sperm production (oligospermia, azoospermia), ejaculation disorders, infections, hormonal imbalances, or exposure to toxins and certain genetic factors [4]. Phytotherapy is a therapeutic approach based on the use of plants or plant extracts to prevent, relieve, or treat various conditions. It relies on the biologically active properties of natural plant compounds, such as alkaloids, flavonoids, tannins, and saponins [5].

Several plants have been studied for their potential impact on male infertility, as well as for the role of their essential oils in improving sperm quality [6]. These plants, belonging to different botanical families, may exert beneficial effects on male fertility. *Mentha suaveolens *(apple mint)* *and *Rosmarinus officinalis *(rosemary) are rich in phenolic compounds with antioxidant properties that can protect spermatozoa from oxidative stress, a major factor in male infertility. Oxidative stress, caused by excessive reactive oxygen species (ROS), can induce lipid peroxidation of the sperm plasma membrane, impair mitochondrial adenosine triphosphate (ATP) production necessary for flagellar movement, and damage sperm DNA, leading to increased DNA fragmentation and reduced motility [7]. Because spermatozoa have limited antioxidant defenses, natural antioxidants from these plants may help preserve sperm function and genetic integrity [7].

The essential oil of *Rosmarinus officinalis *has demonstrated significant antioxidant activity, helping to protect sperm cells from oxidative damage and improve their vitality [8]. *Lippia citriodora *(lemon verbena) also exhibits anti-inflammatory and antioxidant effects that may improve sperm quality, although studies on its direct impact remain limited [9]. Thus, these plants, through their antioxidant, hormonal, and protective effects, could play a role in improving male fertility, and the integration of their essential oils into the management of male infertility, particularly in the context of assisted reproductive technology, appears to be a promising approach, although further clinical studies are needed to confirm their efficacy [10]. Our study aimed to evaluate the effect of *in vitro* supplementation with essential oils on human sperm motility and vitality, as well as its impact on sperm DNA integrity, assessed through the DNA fragmentation index (DFI) and the sperm chromatin decondensation index (SDI). These parameters are crucial for better understanding the potential of essential oils in improving sperm quality and their role in the treatment of male infertility.

## Materials and methods

Ethical standards

The study was approved by the ethics committee of the Faculty of Medicine and Pharmacy, Mohammed VI University of Health and Sciences, Casablanca, Morocco (Biomedical Research Ethics Committee, reference number CE/UM6SS/10/24). All participants provided written informed consent after being fully informed about the purpose and procedures of the study.

Plant material and hydrodistillation

**Figure 1:**
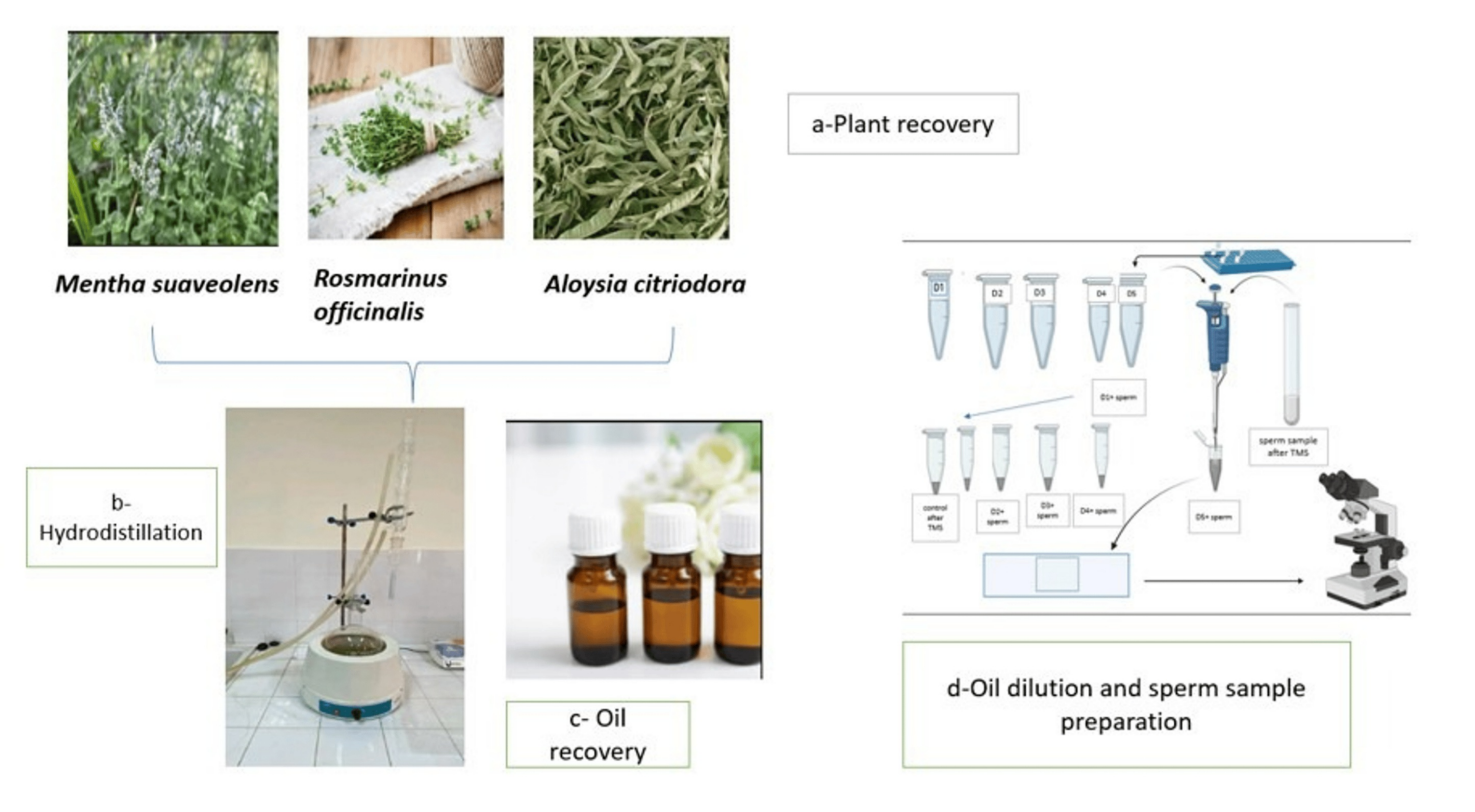
Graphical abstract illustrating plant material processing, essential oil extraction, and sperm sample preparation (a) Plant material collection and preparation, (b) Hydrodistillation process using a Clevenger apparatus. (c) Essential oil collection and recovery. (d) Essential oil dilution and sperm sample preparation. Image created by the authors using BioRender (BioRender, Toronto, Canada).

The essential oils used in this study were extracted from three plant species: *Mentha suaveolens *(leaves), *Rosmarinus officinalis *(leaves), and *Lippia citriodora *(leaves). For the extraction, the powdered leaves were combined with distilled water in a round-bottom flask and subjected to hydrodistillation using a Clevenger apparatus. After 3 hours, the essential oil was collected and stored away from light (Figure 1) [11,12].

Data collection

This *in vitro *experimental study was conducted from January to October 2024 at the Laboratory of Medical Analysis and Reproductive Biology, “Labomac,” in Casablanca, Morocco. A total of 240 men undergoing infertility evaluation were consecutively enrolled. For each participant, the semen sample was divided into multiple aliquots, each of which was exposed to a specific concentration of essential oil as well as to control conditions. Therefore, all experimental groups were tested using aliquots from the same initial sample, allowing direct comparison between treatments while controlling for inter-individual variability. Each participant contributed only one independent semen sample. Eligible participants were aged 25-45 years and had semen parameters consistent with either normozoospermia or asthenozoospermia, according to the 2021 World Health Organization (WHO) reference values. Men with oligozoospermia, azoospermia, genital tract infections, or chronic systemic diseases were excluded.

The study population was divided into two equal subgroups. Group A (n = 120) consisted of normozoospermic samples (sperm concentration ≥ 16 × 10⁶/mL and progressive motility ≥ 30%), whereas Group B (n = 120) comprised asthenozoospermic samples (sperm concentration ≥ 16 × 10⁶/mL and progressive motility < 30%). Semen was obtained by masturbation after 2 to 5 days of sexual abstinence. Following collection, the samples were liquefied at 37 °C in a controlled atmosphere containing 5% CO₂ to stabilize pH and simulate physiological conditions, thus preserving sperm function and allowing accurate evaluation. All analyses and processing steps followed the most recent WHO guidelines [1].

Preparation of dilutions

The essential oils were diluted in a 0.2% (w/v) DMSO solution. Serial dilutions were performed to obtain concentrations ranging from 10 µg/µl to 80 µg/µl. To control for potential solvent effects, a DMSO-only control group (Control 2) was included, consisting of the preparation medium supplemented with 0.2% DMSO but without essential oils. This control ensured that any observed effects on sperm motility or vitality were attributable to the essential oils themselves rather than the solvent.

Sample preparation

After 30 minutes of semen liquefaction, samples from normozoospermic and asthenozoospermic donors were processed using discontinuous density gradient centrifugation with PureSperm® 40/80 (Nidacon, Mölndal, Sweden) according to the manufacturer’s instructions. The resulting sperm pellets were washed twice and resuspended in human tubal fluid medium supplemented with 0.5% human serum albumin. Each sample was then divided into twelve equal aliquots in 1.5 mL Eppendorf tubes. One aliquot from each sample served as a negative control, while the remaining aliquots were incubated with essential oils at concentrations ranging from 10 µg/µL to 80 µg/µL.

Sperm parameter analysis

Motility Assessment

Sperm motility was evaluated using a computer-assisted semen analysis (CASA) system. For each measurement, a 10 μL sample of the semen-essential oil mixture (or control) was placed on a 20 μm Makler chamber. The evaluation was carried out immediately using the Hamilton-Thorne HTM IVOS Analyzer (Hamilton-Thorne Biosciences, Beverly, USA) [13]. Progressive motility was calculated by combining categories a (rapid progressive) and b (slow progressive), expressed as a percentage of the total sperm count. To obtain a detailed overview of sperm movement, several kinetic parameters were recorded: curvilinear velocity (VCL), straight-line velocity (VSL), average path velocity (VAP), amplitude of lateral head displacement (ALH), beat cross frequency (BCF), linearity (LIN), and straightness (STR).

Vitality Assessment

Sperm vitality was evaluated using a 2% eosin stain. Equal volumes of semen and eosin were mixed, and a thin smear was spread onto a glass slide and left to air-dry. The slides were examined under a light microscope as soon as they were dry [14].

DNA Fragmentation Assessment

Sperm DNA integrity was measured using the TUNEL (Terminal deoxynucleotidyl transferase dUTP Nick-End Labeling) assay with a commercial kit (Roche Diagnostics, Lewes, United Kingdom). This method detects DNA fragmentation by marking free 3′-OH ends. Samples were washed twice in phosphate-buffered saline (PBS) and adjusted to a concentration of 2 × 10⁷ cells/mL. The sperm cells were then fixed in 2% formaldehyde for one hour at room temperature, followed by additional PBS washes and centrifugation steps. The TUNEL reaction was carried out according to the manufacturer’s protocol, and stained cells were observed using a fluorescence microscope (Nikon Eclipse 80i; Nikon, Tokyo, Japan). Reference ranges for the DNA fragmentation index (DFI) were defined as: <15% (normal), 15-30% (intermediate), and >30% (high fragmentation) [15,16]. For the sperm chromatin decondensation index (SDI), values ≤25% were considered normal, while values >25% indicated increased chromatin decondensation.

Sperm Chromatin Decondensation Index (SDI) Using Aniline Blue

To evaluate SDI, sperm chromatin was stained with a 0.1% aniline blue solution, which selectively binds to lysine-rich histones present in immature or poorly condensed chromatin. Smears were incubated with the stain for 15 minutes, rinsed, and air-dried. Slides were then examined under a fluorescence microscope, allowing the identification of spermatozoa with residual histones (positive staining) versus mature sperm with properly condensed chromatin (unstained). The percentage of spermatozoa exhibiting positive staining was calculated as the SDI. This method provides an indirect measure of sperm chromatin maturity and decondensation [15].

Statistical analysis

All results were expressed as mean ± standard deviation (SD). The Shapiro-Wilk test was used to confirm the normality of the data. Differences among three or more groups were analyzed using one-way ANOVA, followed by Tukey’s post hoc test when appropriate. For comparisons involving only two groups, the Student’s t-test was applied. Categorical data were compared using the chi-square test or Fisher’s exact test. All analyses were performed using IBM SPSS Statistics version 27.0.1 (IBM Corp., Armonk, USA). A p-value below 0.05 was considered statistically significant.

## Results

Comparison of the characteristics of the two studied groups

The study included two groups: normozoospermia and asthenozoospermia. No statistically significant differences were found between the two groups in terms of mean age (45.79 ± 1.7 vs. 46.26 ± 1.38 years; t(238) = -2.35, p = 0.06), alcohol consumption (56% ± 0.446 vs. 64% ± 0.487; t (238) = -1.30, p = 0.063), or infertility type, whether primary (63.2% ± 0.487 vs. 55.1% ± 0.502; t(238) = -0.93, p = 0.092) or secondary (22.4% ± 0.421 vs. 63.2% ± 0.487; t(238) = -1.34, p = 0.071). In contrast, significant differences were observed for smoking (60% ± 0.49 vs. 70% ± 0.496; t (238) = -1.56, p = 0.03) and the presence of varicocele (19.16% ± 0.19 vs. 74.16% ± 0.43; t (238) = -12.91, p = 0.02) (Table 1).

**Table 1:** Inclusion criteria and comparison of study groups The data are expressed as mean ± standard deviation. Comparisons between groups were carried out using Student’s t-test. A p-value <0.05 was considered statistically significant (**p < 0.01 - indicates a stronger level of significance).

Patient characteristics	Normozoospermia	Asthenozoospermia
Age (years)	45.79 ± 1.7	46.26 ± 1.38
Smoking (presence, yes/no)	60% ± 0.49	70% ± 0.496**
Alcohol consumption (presence)	56% ± 0.446	64% ± 0.487
Primary infertility (presence)	63.2% ± 0.487	55.1% ± 0.502
Secondary infertility (presence)	22.4% ± 0.421	63.2% ± 0.487
Varicocele (presence)	19.2% ± 0.19	74.2% ± 0.43**

Impact of *in vitro *essential oil supplementation on human sperm motility and vitality

**Figure 2:**
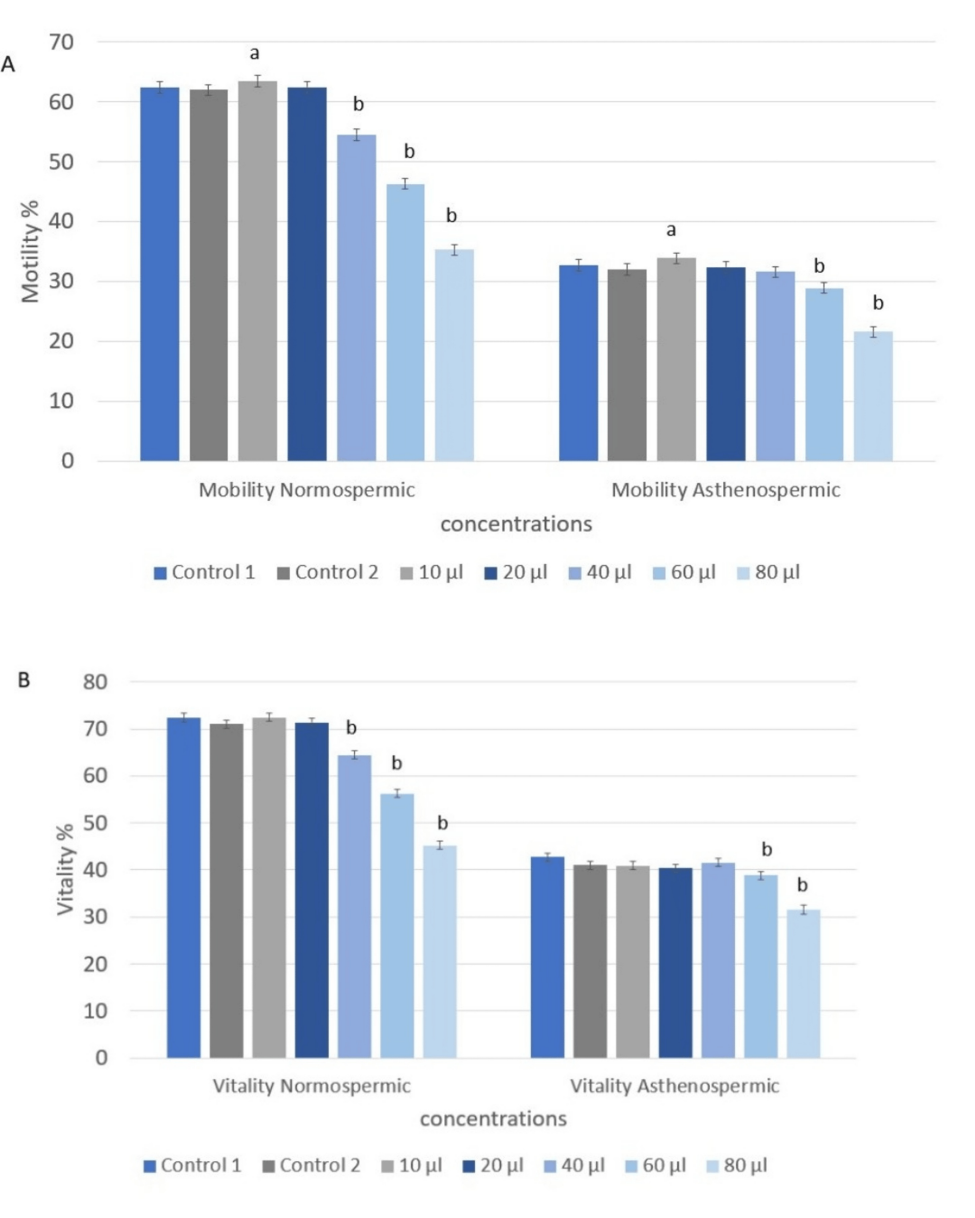
Effect of Mentha suaveolens essential oil on sperm motility and vitality in normospermic and asthenospermic samples. A. The effect of *Mentha suaveolens *essential oil on sperm motility in normospermic and asthenospermic samples. B. The effect of *Mentha suaveolens *essential oil on sperm vitality in normospermic and asthenospermic samples. Analysis was performed using ANOVA. Statistical significance is indicated as follows: a = p < 0.05, b = p < 0.001.

The effect of *Mentha suaveolens *essential oil supplementation on sperm motility and vitality is shown in Figure 2. Figure 2 demonstrates a significant improvement in sperm motility following the addition of the essential oil at a concentration of 10 µg/µl. In the normozoospermic group, motility increased significantly (70% vs. 65%, F (1,118) = 33.3, p < 0.001) (Figure 2A), whereas vitality showed a slight increase that was not statistically significant (76% vs. 74.5%, F (1,118) = 3.0, p = 0.08) (Figure 2B). Similarly, in the asthenozoospermic group, motility improved (35.1% vs. 33.5%, F (1,118) = 1.39, p = 0.07), while vitality did not change significantly (44.93% vs. 44.9%, F (1,118) = 0.001, p = 0.09).

**Figure 3:**
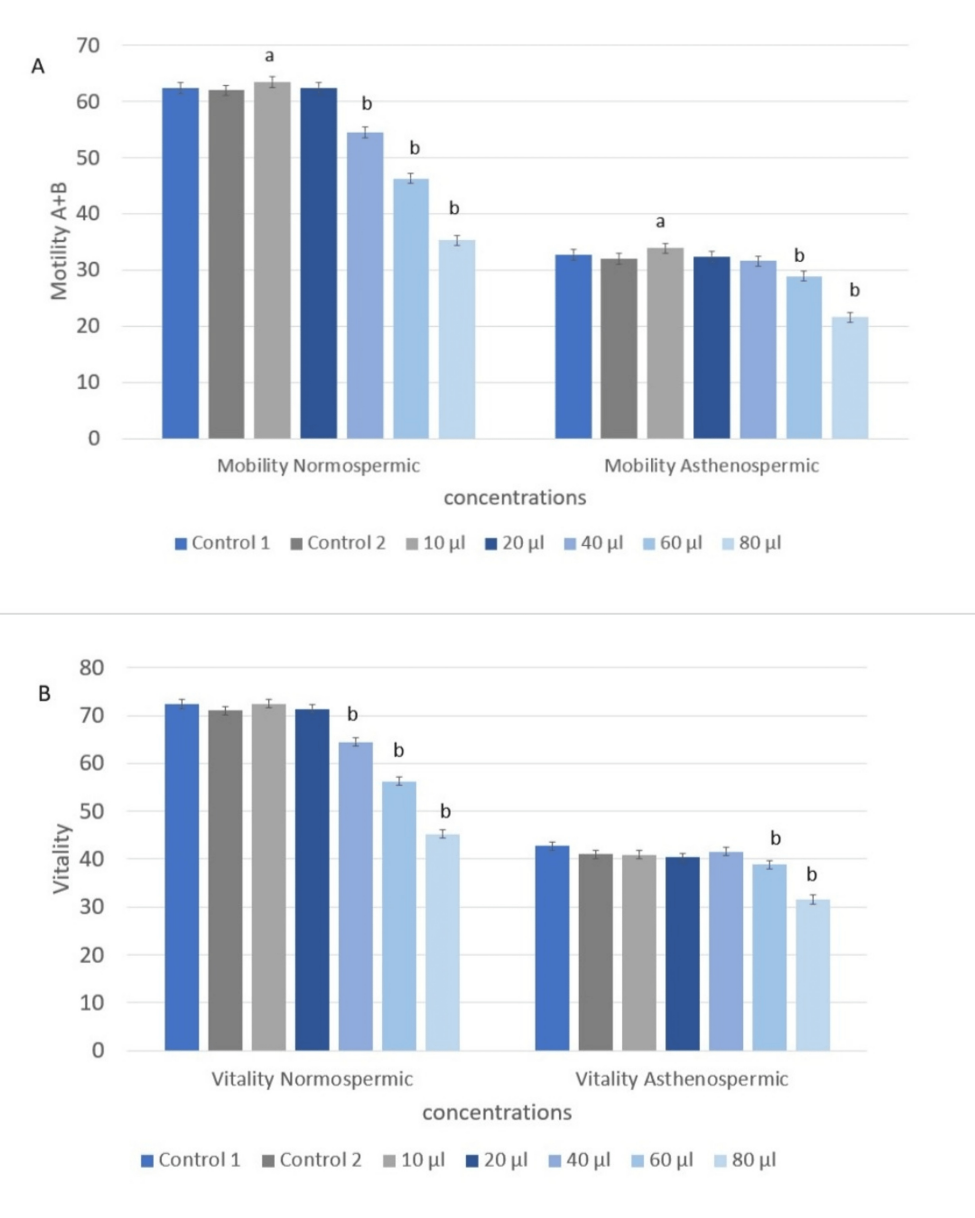
Effect of Rosmarinus officinalis essential oil on sperm motility and vitality in normospermic and asthenospermic samples. A. The effect of *Rosmarinus officinalis *essential oil on sperm motility in normospermic and asthenospermic samples. B. The effect of *Rosmarinus officinalis *essential oil on sperm vitality in normospermic and asthenospermic samples. ANOVA was used to analyze the data. Statistical significance is denoted as a: p < 0.05 and b: p < 0.001.

Figure 3 illustrates the impact of *Rosmarinus officinalis *essential oil supplementation on sperm motility and vitality. The results show an improvement in sperm motility following exposure to the essential oil at a concentration of 10 µg/µl. In normozoospermic samples, motility increased significantly (64% vs. 62.38%, F (1,118) = 2.40, p = 0.01) (Figure 3A), whereas vitality showed a slight, non-significant increase (72.4% vs. 72.38%, F(1,118) = 0.0004, p = 0.09) (Figure 3B). Similarly, in asthenozoospermic samples, motility improved significantly (35.9% vs. 32.7%, F(1,118) = 5.24, p = 0.02), while vitality did not change significantly (43% vs. 42.7%, F(1,118) = 0.046, p = 0.08).

**Figure 4:**
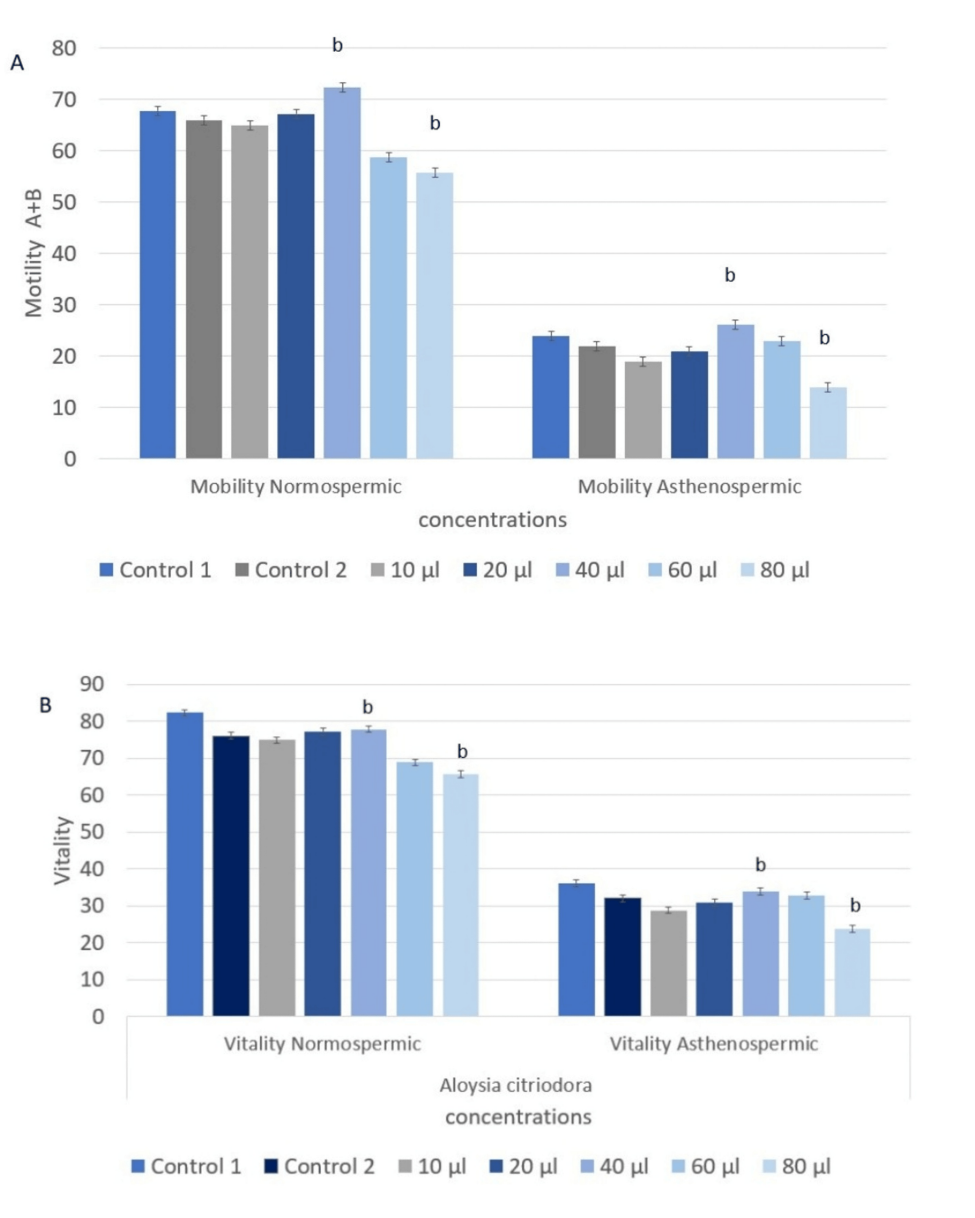
Effect of Lippia citriodora essential oil on sperm motility and vitality in normospermic and asthenospermic samples. A. The effect of *Lippia citriodora *essential oil on sperm motility in normospermic and asthenospermic samples. B. The effect of *Lippia citriodotra *essential oil on sperm vitality in normospermic and asthenospermic samples. Analysis was performed using ANOVA. Statistical significance is indicated as a: p < 0.05; b: p < 0.001.

The impact of *Lippia citriodora *essential oil supplementation on sperm motility and vitality is shown in Figure 4. Figure 4 demonstrates a significant improvement in both parameters following the addition of the essential oil at a concentration of 40 µg/µl. In the normozoospermic group, motility and vitality increased (72.24% vs. 67.8%, F(1,118) = 20.4, p < 0.001), (77.8% vs. 74.82%, F(1,118) = 9.21, p < 0.001). Similarly, a significant improvement was observed in the asthenozoospermic group (26.06% vs. 23.83%, F(1,118) = 4.52, p < 0.001, and 34% vs. 33.8%, F(1,118) = 0.036, p < 0.001, respectively).

Influence of essential oils supplementation on DFI and SDI in normozoospermic and asthenozoospermic groups

**Figure 5:**
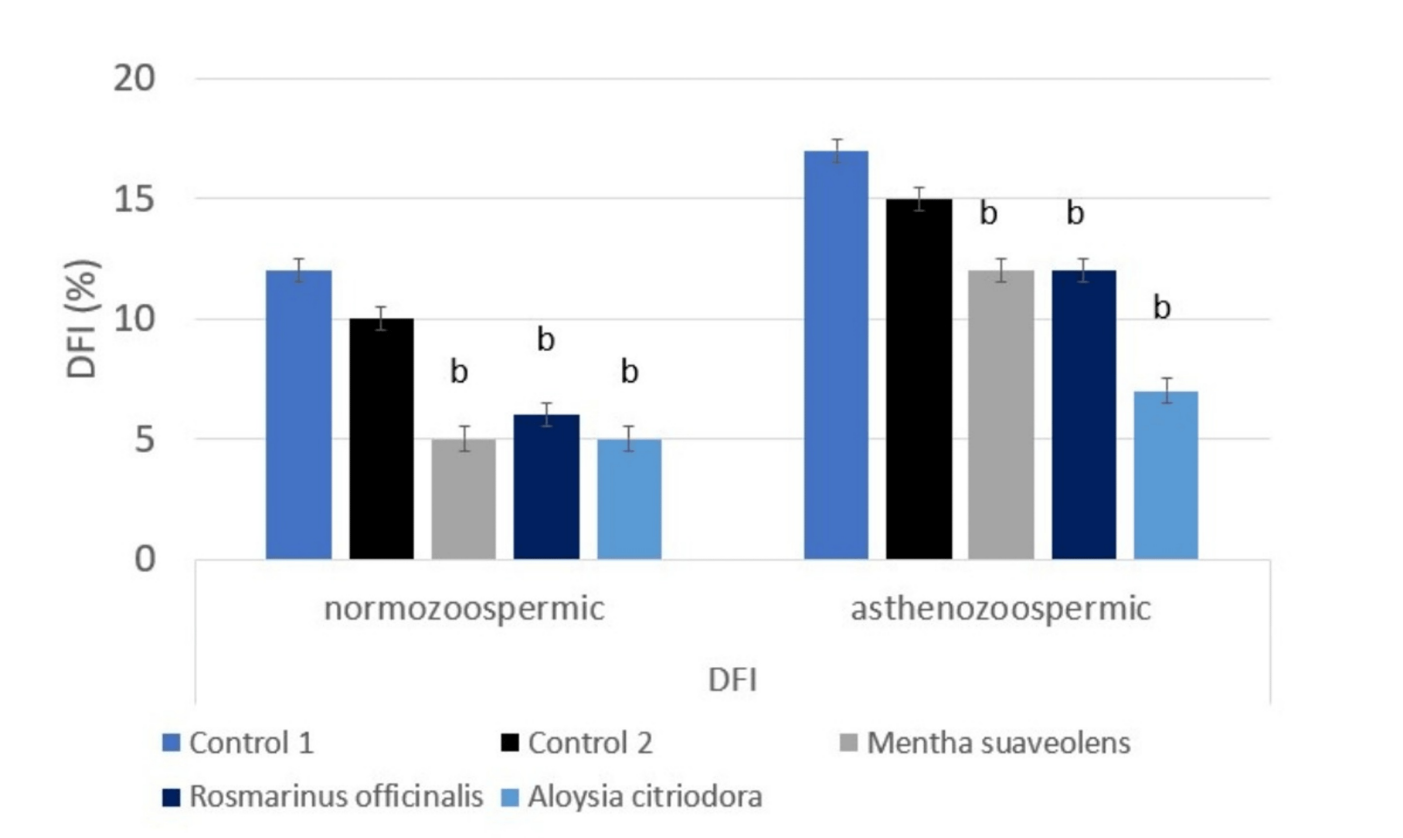
Assessment of DNA fragmentation index (DFI) in human sperm after in vitro essential oil treatment Analysis was performed using ANOVA. Statistical significance is indicated as b: p < 0.001.

**Figure 6:**
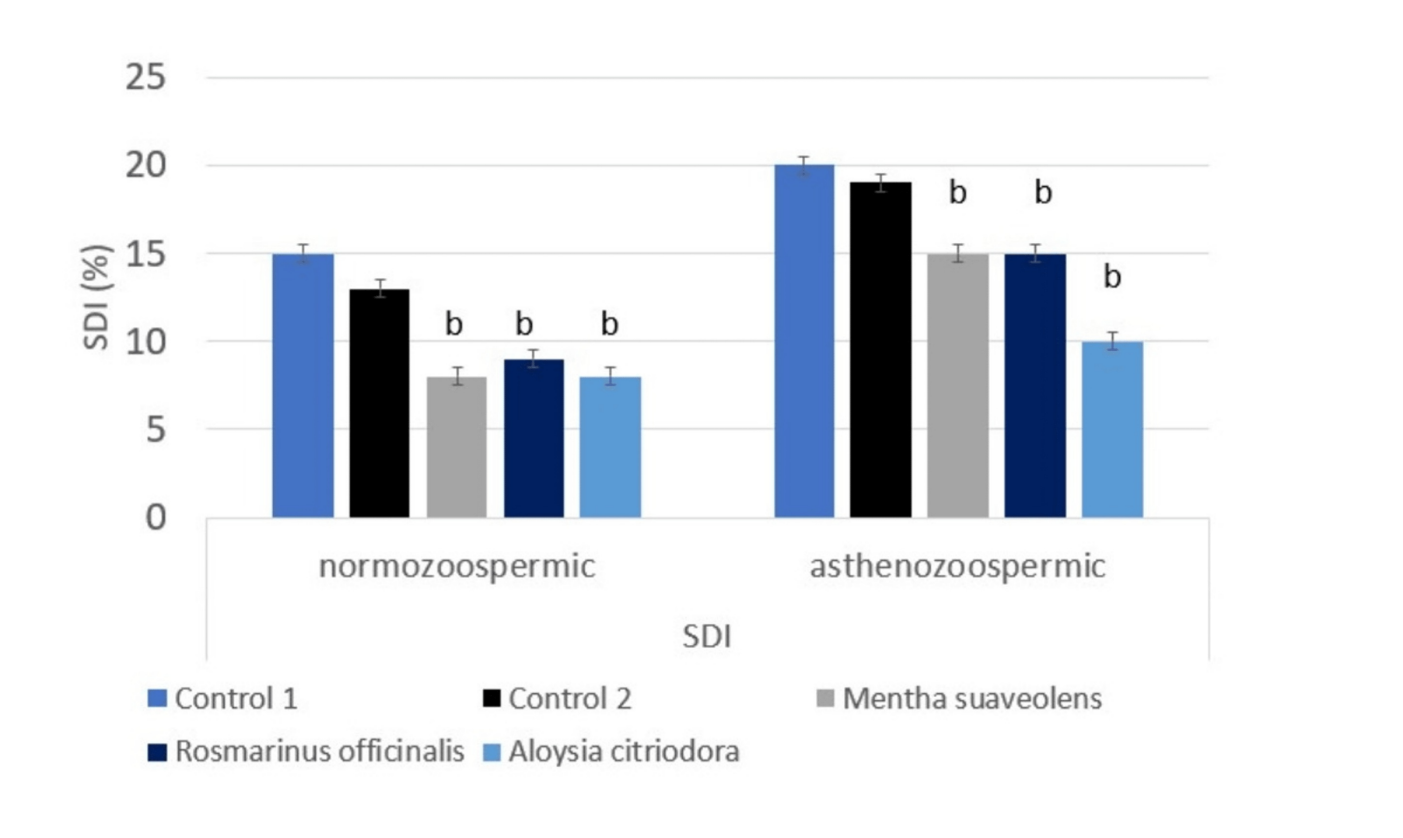
Evaluation of sperm chromatin decondensation index (SDI) in response to in vitro essential oil supplementation ANOVA was used to analyze the data. Statistical significance is denoted as b: p < 0.001.

The proportion of DNA-fragmented spermatozoa was significantly reduced in both normozoospermic and asthenozoospermic samples following treatment with *Mentha suaveolens*, *Rosmarinus officinalis*, or *Lippia citriodora*. In normozoospermia, fragmentation decreased from 12% in controls to 5-6% across treatments (F (1,118) ≈ 25, p < 0.001). In asthenozoospermia, fragmentation declined from 17% in controls to 7-12% (F(1,118) ≈ 22, p < 0.001) (Figure 5). Similarly, the sperm chromatin decondensation index was significantly improved in treated groups. In normozoospermia, the sperm chromatin decondensation index decreased from 15% in controls to 8-10% after treatment (F (1,118) ≈ 18, p < 0.001), while in asthenozoospermia it declined from 20% to 12-15% (F (1,118) ≈ 20, p < 0.001) (Figure 6).

## Discussion

Phytotherapy offers a promising approach in managing male infertility by harnessing the bioactive properties of various medicinal plants [9]. Several studies have demonstrated that certain plant extracts, rich in antioxidants, flavonoids, and anti-inflammatory compounds, can enhance sperm quality, particularly by reducing oxidative stress [17]. Our study was designed to assess the impact of *in vitro *supplementation with three essential oils: *Mentha suaveolens *(apple mint), *Rosmarinus officinalis *(rosemary), and *Lippia citriodora *(lemon verbena) at different concentrations on human sperm quality. Specifically, we aimed to assess their beneficial and adverse effects on sperm quality. These parameters are essential for a better understanding of the potential of essential oils in improving sperm quality and their possible role in the treatment of male infertility.

The essential oil of *Mentha suaveolens*, known for its antimicrobial, antioxidant, and anti-inflammatory properties, revealed a dose-dependent effect at the optimal concentration of 10 µg/µL. Motility significantly increased in normozoospermic samples (70% vs. 65%, p < 0.001) and slightly in asthenozoospermic samples (35.1% vs. 33.5%, p = 0.07), with overall vitality maintained in both groups (76% vs. 74.5%, p = 0.08; 44.93% vs. 44.9%, p = 0.09). Alongside a significant reduction in DNA fragmentation (12% vs 5% in the normozoospermic group and 17% vs 12% in the asthenozoospermic group, p < 0.001) and a significant reduction in sperm chromatin decondensation index (15% vs 8% in the normozoospermic group and 20% vs 15% in the asthenozoospermic group, p < 0.001). However, higher concentrations (20 µg/µL) caused a marked decrease in motility and vitality, indicating cytotoxicity, as the sperm parameters declined proportionally with increasing doses. Previous studies have reported that *Mentha suaveolens *contains bioactive compounds, especially monoterpenes and sesquiterpenes, such as pulegone and menthone, which contribute to its antioxidant and protective activities. While chromatographic analyses reported in the literature confirm these constituents, we did not perform gas chromatography - mass spectrometry (GC-MS)** **or chromatographic characterization on the oils used in the present study. Future studies could include GC-MS profiling to identify and quantify specific bioactive components responsible for the observed effects [18,19]. Additionally, anticorrosive properties of *Mentha suaveolens *essential oil have been reported for metal surface protection, acting as an effective inhibitor on mild steel in acidic environments [19].

In *Rosmarinus officinalis*, which is rich in 1,8-cineole, α-pinene, and camphor [20], a similar dose-dependent pattern was observed. At 10 µg/µL, motility significantly increased in normozoospermic (64% vs. 62.38%, p = 0.01) and asthenozoospermic samples (35.9% vs. 32.7%, p = 0.02), with vitality (72.4% vs. 72.38%, p = 0.09) in the normozoospermic group (43% vs. 42.7%, p = 0.08). asthenozoospermic group. Alongside a significant reduction in DNA fragmentation (12 vs 6%, normozoospermic group and 17 vs 12% asthenozoospermic group (p<0.001), and a significant reduction in sperm chromatin decondensation index (15 vs 9%, normozoospermic group and 20 vs 15% asthenozoospermic group (p<0.001). At the concentration of 40 µg/µL, higher doses resulted in a marked reduction of these beneficial effects. Prior studies demonstrated rosemary’s capacity to improve sperm motility, notably in poultry sperm storage, where 0.2 µL/mL enhanced motility and vitality over 72 hours [21]. Dietary supplementation also improved sperm parameters and antioxidant status in rabbits [22] and reduced oxidative stress while protecting spermatogenesis in diabetic rats [23].

*Lippia citriodora*, rich in citral, limonene, and verbascoside [24], displayed consistent and significant improvement in motility at 40 µg/µL, increasing normozoospermic motility (72.24% vs. 67.8%, p <0.001) and asthenozoospermic motility (26.06% vs. 23.83%, p < 0.001), improvement with vitality in both groups (77.8% vs. 74.82%, p < 0.001; 34% vs. 33.8%, p <0.001), and reduced DNA fragmentation (12 vs 5%, group A and 17 vs 7% asthenozoospermic group, p<0.001), and DNA denaturation (15 vs 10% normozoospermic group and 20 vs 12% asthenozoospermic group, p<0.001) without the toxicity observed at higher concentrations in the other oils. This plant is known for its neuroprotective, antimicrobial, and calming effects [24,25], and dietary supplementation improved sperm motility, morphology, and vitality in brown hares and rabbits [26,27]. Encapsulation in niosomes has also been shown to enhance the preservation of its bioactive compounds [28].

*Lippia citriodora*, *Rosmarinus officinalis*, and *Mentha suaveolens *are aromatic medicinal plants whose essential oils contain a wide array of bioactive compounds with potent antioxidant and cytoprotective effects, making them promising candidates for improving sperm motility and vitality *in vitro *[21,29]. The increase in vitality appears closely related to their capacity to preserve plasma membrane integrity, prevent lipid peroxidation, and support mitochondrial function, key factors for sperm vitality [23]. For instance, *Lippia citriodora*, rich in verbascoside and phenylpropanoids, effectively scavenges reactive oxygen species (ROS), reducing oxidative stress that damages membranes and induces apoptosis [27]. Similarly, *Rosmarinus officinalis*, via rosmarinic acid, carnosol, and carnosic acid, protects mitochondrial membranes and limits pro-apoptotic factor release, supporting ATP production essential for motility and longevity [21].

*Mentha suaveolens*, containing monoterpenes like pulegone and carvone, helps maintain intracellular enzymatic activity and reduces oxidative damage to DNA and cytoskeletal elements, thereby enhancing overall sperm vitality [29]. However, at higher concentrations, these essential oils may exert cytotoxic effects, leading to membrane destabilization, mitochondrial dysfunction, and a subsequent decline in motility and vitality, highlighting a dose-dependent balance between antioxidant protection and cellular toxicity [23]. Importantly, these essential oils do not revive dead spermatozoa; rather, they delay cellular degradation and preserve the function of already viable spermatozoa, leading to improved motility and vitality parameters [21,29].

The present study has several limitations. It was conducted *in vitro*, so the results may not directly translate to *in vivo *conditions. Additionally, the essential oils were not characterized using GC-MS or other chromatographic techniques, which limits the identification of specific bioactive components responsible for the observed effects. Future studies should address these limitations by including larger cohorts, performing detailed chemical profiling of the oils, and utilizing *in vivo *models to further confirm their therapeutic potential in enhancing male fertility.

## Conclusions

In conclusion, our study highlights the promising potential of phytotherapy, particularly essential oils, in improving sperm quality by modulating oxidative stress, DNA integrity, and other functional parameters. Various plant extracts and essential oils have demonstrated beneficial effects on sperm motility, vitality, and DNA integrity, mainly due to their antioxidant, anti-inflammatory, and hormonal properties. These effects are highly concentration dependent, and it is crucial to identify the specific active molecules responsible and determine the correct non-cytotoxic doses. While these natural compounds present interesting therapeutic prospects, further clinical research is necessary to assess their safety, effectiveness, and optimal application in the management of male infertility, especially in conjunction with assisted reproductive technologies (ART). Practical considerations for clinical translation include evaluating potential cytotoxicity at different concentrations, ensuring compatibility with standard sperm preparation media, and complying with regulatory requirements for the use of natural compounds in ART laboratories. Additional studies are also needed to examine long-term effects on sperm function, fertilization outcomes, and embryo development.
